# Cardiac and respiratory arrest in a 12‐year‐old girl with acute permethrin oral toxicity: A case report

**DOI:** 10.1002/ccr3.5245

**Published:** 2022-01-11

**Authors:** Hesam Adin Atashi, Hamid Zaferani Arani, Felicia Agatha, Seyyed Mojtaba Ghorani, Mahya Sadat Teimouri Khorasani, Masoumeh Moalem

**Affiliations:** ^1^ School of Medicine Tehran Medical Sciences Islamic Azad University Tehran Iran; ^2^ Tzu Chi Secondary School PIK DKI Jakarta Indonesia; ^3^ Department of Emergency Tehran Medical Sciences Islamic Azad University Tehran Iran

**Keywords:** acute toxicity, cardiac arrest, ingestion, insecticide, permethrin poisoning, pyrethroid, respiratory arrest

## Abstract

Permethrin (PER) is widely employed as the most frequently used type I synthetic pyrethroid insecticide. Despite its worldwide application, reports of pediatric toxicity following permethrin administration are scarce. The present report describes the case of a 12‐year‐old girl with cardiac and respiratory arrest resulting from self‐induced oral toxicity by permethrin.

## Graphical abstract

We presented a 12‐year‐old girl intoxicated orally with permethrin. Toxicity symptoms can vary from headache and dyspnea to metabolic acidosis and cardiac and respiratory arrest, which lead to hypoxic brain damage and death, as occurred in this case.

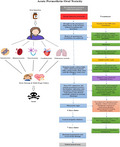

## 1 INTRODUCTION

Permethrin (C_21_H_20_Cl_2_O_3_) is the most frequently used type I synthetic pyrethroid insecticide, which is extensively used worldwide.^1^ However, the expanded use of permethrin might cause various toxic effects on humans, including neurotoxicity, immunotoxicity, cardiotoxicity, hepatotoxicity, digestive system toxicity, and cytotoxicity, and also reproductive, genotoxic, and hematotoxic effects.^2^ According to the European Chemicals Agency (ECHA), permethrin may be fatal if swallowed and enters airways (Aspiration hazard, Category 1 H304).^3^ The main molecular targets of synthetic pyrethroids are voltage‐dependent sodium channels (VDSC); they bind to these channels and delay the inactivation of sodium channels, resulting in neurotoxic effects and, ultimately, death.^4^ The main type of exposure is dermal absorption, while its major neurotoxic mechanisms include oxidative stress, inflammation, neuronal cell loss, and mitochondrial dysfunction.^5^ Permethrin is administered as the first‐line therapy for scabies in pediatric patients older than 2 months in many countries, which may lead to accidental poisoning in few cases, especially among children.^6^

Despite its global application, there have been scarce reports of pediatric toxicity following permethrin administration. The present report describes the case of a 12‐year‐old girl with cardiac and respiratory arrest resulting from self‐induced oral toxicity by permethrin.

## 2 CASE PRESENTATION

A 12‐year‐old Afghan girl, with no previous medical problem, drank an unknown insecticide covertly at home. Two hours after ingestion, symptoms of headache, vertigo, vomiting, dyspnea, and confusion presented at home, and after 20 min, she was taken to the emergency room with neither breathing signs nor a heartbeat. She was immediately transferred to the cardiopulmonary resuscitation (CPR) room. A few minutes following CPR, spontaneous circulation was returned achieved with chest compression, bag valve mask, artificial ventilation, and two doses of 1 mg intravenous epinephrine. Moreover, gastric lavage and activated charcoal administration were performed after intubation. Then, she was volume resuscitated with intravenous normal saline and connected to the mechanical ventilator after being transferred to the intensive care unit (ICU) ward. Following admission to ICU, she presented excessive lacrimation, salivation, and myoclonic muscle twitches. Physical examinations indicated pinpoint pupils with no reflex to light, downward plantar reflex, normal lung sounds, tachycardia, normal blood pressure, and respiratory rate. She was in a deep coma in the interim with a Glasgow coma score of 3, while electrocardiography showed sinus tachycardia. Computed tomography (CT) scans of patient's head and chest, along with abdominal X‐rays, were normal. Even though laboratory test results showed metabolic acidosis in arterial blood gas and increased blood levels of creatine kinase myocardial band (CK‐MB), other indicators were normal (Table 1).

**TABLE 1:** Results of the laboratory tests for the patient

Toxicology urine laboratory	Result	Reference
Amphetamine	Negative	–
Methamphetamine	Negative	–
Cocaine	Negative	–
Morphine	Negative	–
Methadone	Negative	–
Barbiturates	Negative	–
pH	5	–
Tetrahydrocannabinol	Negative	–
Tricyclic antidepressants	Negative	–
Permethrin	Positive*	–
Buprenorphine	Negative	–
Tramadol	Negative	–
Benzodiazepines	Negative	–

Lab Data	Result	Reference
Blood Culture X2	(1&2) No Growth After 48 h	–
Urine Culture X2	No Significant Uropathogens Isolated	–
White Blood Cells	11,800 count*	4000–11,000
Red Blood Cells	4.84 mill/cumm	3.9–5.6
Hemoglobin	13 g/dl	11–16.5
Platelets	243,000 count	150,000–450,000
Creatine Kinase	157 IU/L	24–170
Troponin	0.012 ng\ml	<0.04
CK‐MB	99 IU/L*	<24
Blood Urea	21 mg/dl	15–40
Creatinine	0.8 mg/dl	0.6–1.4
Arterial Blood Gas pH	6.99*	7.35–7.45
PCO2	37.9*	40
HCO3	9.3 mmol/L*	24
3 Days After Admission		
Plasma Copeptin	2.3 pmol/L	≥3.6
Urine Specific Gravity	1.003*	1.005–1.030
Serum Sodium Level	159 meq/L*	135–145
Blood Glucose Level	126 mg/dl	<140
AST	41 IU/L*	<31
ALT	22 IU/L	<31
Alkaline Phosphatase	309 IU/L*	64–306

Abbreviations: ALT, Alanine Transaminase (asterisk marks for the abnormal findings); AST, Aspartate Transaminase; CK‐MB, Creatine Kinase‐Myocardial Band; HCO3, Hydrogen Carbonate; PCO2, Pressure of Carbon Dioxide; PH, Potential of Hydrogen.

In the ICU, her muscle twitches and metabolic acidosis were controlled with 5 mg intravenous atracurium and 5 mg levetiracetam and bicarbonate sodium vials, respectively. In the days following hospitalization, the patient had normal blood pH in arterial blood gas analysis samples. Moreover, in the ICU, she was administered two doses of 1 mg intravenous atropine to treat muscarinic symptoms; however, there was no therapeutic response. The patient continued to be comatose without spontaneous breathing, pupils were fixed dilated bilaterally, and central diabetes insipidus was suspected after observing hypernatremia in patient's blood test, which was higher than 4.5 L in the 24‐h urine volume. Central diabetes insipidus became evident after 3 days due to apnea and hypoxic brain damage following the insecticide ingestion. It was diagnosed based on decreased urine specific gravity and abnormal plasma copeptin level.

The chemical analysis of the insecticide bottle showed 10% permethrin, without organophosphates, as initially expected (Table 1). Unfortunately, she passed away after 7 days due to resistant hypotension and severe brain damage.

## 3 DISCUSSION

Pyrethroids impact voltage‐dependent sodium chloride and chloride channels.^7^ However, toxicities are scarce in gastrointestinal, neurologic, and respiratory systems due to this compound.^8^ Pyrethroids are categorized into types I and II, and permethrins are considered type I (ie, the cyclopropane group).^9^ Based on the reported acute toxicity of permethrin, that is, an LD50 of 430–4000 mg/kg for industrial permethrin in rats, it is slightly toxic through the dermal route. The 4‐h inhalation LC50 for the rats was more significant than 23.5 mg/L, indicating practically no inhalation toxicity.^10^, ^11^

Following 4–48 h of the intentional ingestion of pyrethroid, gastrointestinal, neurologic, or pulmonary signs and symptoms will appear, including headache and fatigue as the most common symptoms. However, several fewer common signs and symptoms, such as hepatic and renal dysfunction, are inevitable.^7^ Nausea, vomiting, sore throat, and epigastric pain are symptoms of the oral consumption of pyrethroids.^8^ Permethrin toxicity can show organophosphorus poisoning because of its cholinergic actions, while atropine is not effective to control signs as it experienced in our and other studies.^2^

The deliberate use of a large volume of permethrin by a 12‐year‐old girl with suicidal intent is rare. As confirmed by similar studies, mortality is rare among these patients.^2^, ^9^, ^12^ In particular, most permethrin toxicity cases have occurred through dermal contact, as opposed to our case of pediatric oral permethrin toxicity. Drago et al.^2^ reported cases, in which children inadvertently took low doses of permethrin, and in the study by Yang et al.,^9^ 38 out of 48 patients with a mean age of 49 ± 3 years took permethrin to commit suicide; however, there was only one case of mortality among them.

Some molecular pathways might be involved in cardiotoxicity with pyrethroids. In particular, pyrethroids, such as permethrin, can damage neuronal sodium channels by inducing a persistent steady‐state sodium current within depolarized membranes, leading to cardiac hypotrophy, an increase in calcium release, and the enhancement of the *Nrf2* gene expression levels in older animals. The levels of cytosolic calcium are essential to the cardiac muscle's contractile state. Moreover, permethrin induces oxidative damage to purine bases in cardiac cells.^13^

Permethrin ingestion can lead to apnea and hypoxia, resulting in critical and irreversible organ damage. Brain injury can be progressive, as it occurred in our case, and it can cause many complications.^5^, ^14^ In this report, the patient developed central diabetes insipidus, which deteriorated her condition. Central diabetes insipidus has been found in a variety of disorders that mainly arise from hypothalamus damage. Polyuria and polydipsia occur when more than 80%–90% of the Arginine Vasopressin‐secreting neurons in the supraoptic and paraventricular nuclei are damaged. Patients with hypothalamic neuronal damage from oxidative stress pathways can develop hypoxia and are likely to develop central diabetes insipidus. Extensive destruction can be caused by several acquired pathological processes, including idiopathic conditions.^15^ Atracurium, employed in this study, controlled the muscle twitches and prevented rhabdomyolysis. However, atropine had no positive effect on the patient. Also, in this case, we were suspicious of child abuse and deliberate intoxication, so we investigated the patient's family psychological history, and we performed forensic medicine consult, but there was not any evidence of abuse and all the evidence was in favor of suicide.

**FIGURE 1:**
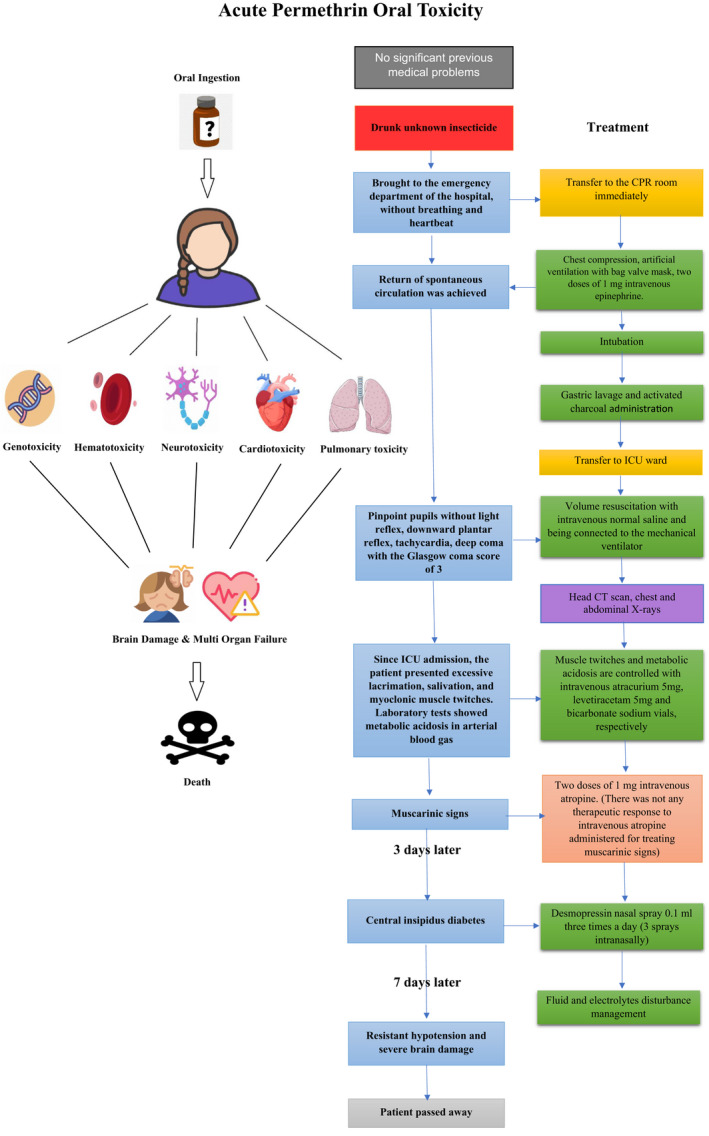
The treatment strategy flowchart for the case

Our primary limitations in this case study were the lack of proper treatment guidelines and an antidote for pediatric permethrin toxicity. Despite the initial uncertainty about the ingested insecticide, the rarity of the case, and poor cooperation from the patient's family, we successfully diagnosed the case in the early stages based on the patient's signs and symptoms and provided the best possible management. We believe that our proposed method can be applied to patients in future cases. However, despite the timely control of the disease, due to the high dosage and volume of the ingested insecticide and the delay in hospitalization, the patient inevitably suffered from irreversible brain hypoxia and went into a coma. The flowchart of the treatment strategy and the process administered for this patient is presented in Figure 1. This flowchart can be used in similar cases to obtain the best treatment results.

Permethrin is a widely used insecticide. However, there are many unmet needs in permethrin toxicity treatment, including the lack of evidence‐based guidelines for most complications, the absence of a specific antidote for poisoning caused by this insecticide, and the ineffectiveness of atropine for muscarinic signs. Moreover, this treatment is mainly supportive. Depending on the amount and dose of permethrin, most common symptoms can vary from headache, dyspnea, and vomiting to metabolic acidosis and cardiac and respiratory arrest, which can lead to hypoxic brain damage and death, as occurred in the reported case.

## CONFLICT OF INTEREST

The authors declare that they have no competing interests.

## AUTHOR CONTRIBUTIONS

HAA and HZA involved in the treatment and clinical management decision making of the patient, obtained consent for the publication, and wrote draft manuscript. SMG, FA, and HZA interpreted clinical data and critically revised the manuscript for important intellectual content. MM and HAA involved in the treatment and clinical management decision making of the patient and critically revised the manuscript.

## ETHICAL APPROVAL

The research obtained approval by the School of Medicine of Tehran Islamic Azad University of Medical Sciences. The participant of the study was volunteer who had given informed consent to the study.

## CONSENT

The parent's patient had given informed written consent to report the individual patient data.

## Data Availability

The clinical documentation of the presented case cannot be made public due to the detailed identifiable information of the patient.
